# A simple aid to fracture reduction in the digit

**DOI:** 10.1308/003588412X13373405385214q

**Published:** 2012-07

**Authors:** C Weddell, A McMurtrie, AK Hamad

**Affiliations:** ^1^Shrewsbury and Telford Hospital NHS Trust,UK; ^2^Robert Jones and Agnes Hunt Orthopaedic Hospital NHS Foundation Trust,UK

## BACKGROUND

We describe a simple method enabling traction to be applied to a digit and providing control of rotation, alignment and length while avoiding inadvertent radiation to the surgeon’s or assistant’s fingers. Furthermore, it provides excellent exposure to the digit, alleviating difficulties in fixation of complex fractures.

## TECHNIQUE

**Figure 1:**
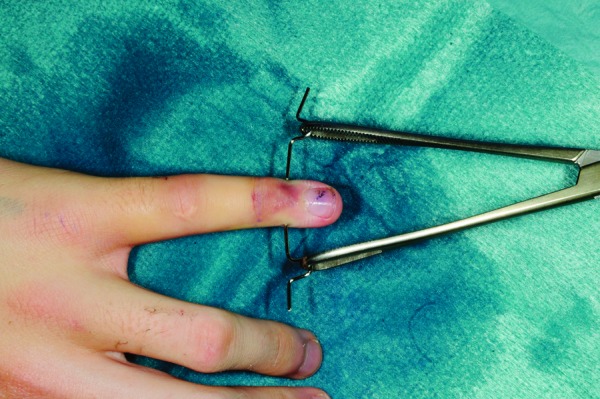
Anteroposterior photograph

**Figure 2:**
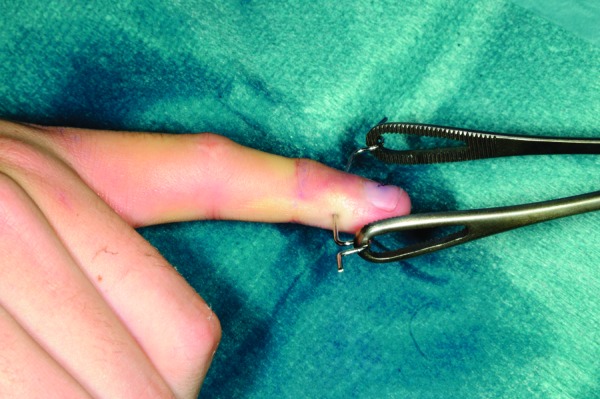
Lateral photograph

**Figure 3:**
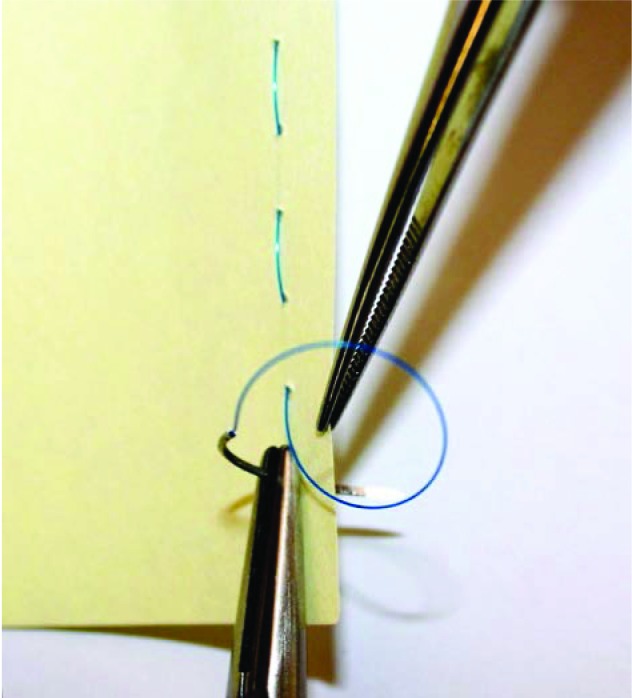
Pre-reduction x-ray

**Figure 4:**
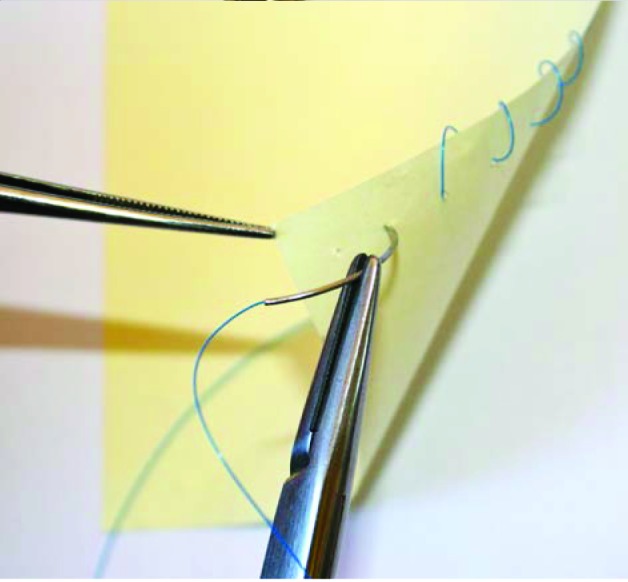
Post-reduction x-ray (with traction)

A 1.1mm K-wire is inserted under fluoroscopic guidance transversely across the base of the distal phalanx of the injured digit. The wire is then bent on either side and the sharp ends trimmed. A Rampley sponge holder is used to hold the wire (Figs 1 and 2). Traction is applied by holding on to the sponge holder to enable ligamentotaxis to reduce the fracture (Figs 3 and 4). Rotational deformity, angulation, length and radial/ulnar deviation can be corrected by applying varying forces. The standard method of fracture fixation can then be performed.

## DISCUSSION

We have found this method extremely useful when fixing fractures of the digits where optimal access to the fracture site is required while continued traction is necessary to maintain reduction. This is particularly the case in fractures of the middle phalanx where alternative methods of applying traction may interfere with access to the fracture site. Although it is not an original principle, we believe that this technique has not been described previously in the literature. We strongly recommend this technique to those involved in the treatment of fractures of the hand.

